# Can We Improve Pregnancy Rates in Hormone Receptor-Positive Breast Cancer After Endocrine Therapy? The Role of Fertility Preservation Beyond Gonadotoxic Therapy

**DOI:** 10.3390/cancers17213498

**Published:** 2025-10-30

**Authors:** Maria Vittoria Luciani, Giorgia Mangili, Enrico Papaleo, Rossella Biancardi, Valeria Stella Vanni, Rossella Masciangelo, Valentina Elisabetta Di Mattei, Massimo Candiani, Raffaella Cioffi

**Affiliations:** 1 Unit of Gynecology and Obstetrics, IRCCS San Raffaele Scientific Institute, 20132 Milan, Italy; mangili.giorgia@hsr.it (G.M.); papaleo.enrico@hsr.it (E.P.); biancardi.rossella@hsr.it (R.B.); vanni.valeria@hsr.it (V.S.V.); masciangelo.rossella@hsr.it (R.M.); candiani.massimo@hsr.it (M.C.); 2 Clinical and Health Psychology Unit, IRCCS San Raffaele Scientific Institute, 20132 Milan, Italy; dimattei.valentina@hsr.it; 3 School of Psychology, Vita-Salute San Raffaele University, 20132 Milan, Italy

**Keywords:** controlled ovarian stimulation, hormonal therapy, oncofertility, breast cancer

## Abstract

**Simple Summary:**

Fertility preservation is safe and effective in breast cancer patients; however, there is a lack of data regarding fertility preservation in hormone receptor-positive breast cancer patients treated exclusively with hormonal therapy. This study aims to assess the feasibility and reproductive outcomes of fertility preservation in this group of patients often excluded from standard oncofertility pathways. We want to emphasize the importance of considering fertility preservation not only to mitigate the gonadotoxic effects of chemotherapy but also to counteract age-related fertility decline during prolonged endocrine therapy. This study may encourage the use of fertility-sparing treatments in patients who are not referred for gonadotoxic therapies, promoting patient adherence to treatment.

**Abstract:**

**Background/Objectives**: Young patients with hormone receptor-positive breast cancer (HR+BC) face an elevated risk of cancer-related mortality, partly due to lower adherence to hormonal therapies for fertilities concerns. This study aims to evaluate fertility preservation in patients receiving hormonal therapy (HT) alone after surgery with or without radiotherapy. **Methods**: This single-center prospective cohort study evaluated BC patients counseled at the Oncofertility Unit of San Raffaele Hospital (2012–2024). **Results**: Of 251 BC patients who received counseling, 39 met the inclusion criteria. Among 33 patients with adequate follow-up, 15 (45.5%) are still under anticancer treatment, 6 (18.2%) completed HT but do not seek pregnancy, and 12 (36.3%) sought pregnancy, of which 9 (75%) conceived. Among the nine patients who conceived, four had completed HT (one became pregnant after thawing her cryopreserved oocytes and three had a spontaneous pregnancy). Five patients who conceived had suspended HT to seek pregnancy, according to the results of the POSITIVE trial. After HT discontinuation, all patients thawed their oocytes: three had a pregnancy with a live birth, while two patients did not conceive, so one of them attempted a new in vitro fertilization cycle achieving pregnancy with a live birth, while the other one had a spontaneous pregnancy. **Conclusions**: Our study highlights the importance of counseling HR+ BC patients candidates for HT alone about the efficacy and safety of fertility preservation. Offering fertility preservation can mitigate the reproductive impact of therapy-related childbearing delays and potentially improve treatment adherence.

## 1. Introduction

Breast cancer (BC) is the most common cancer in women of all ages, and the most diagnosed cancer among women aged 30 to 39 years [1]. In 2023 in Italy, 55,900 new BC diagnoses were made, and 15,500 deaths were estimated [2]. BC incidence continues to rise and according to the GLOBOCAN Cancer Tomorrow prediction tool, the number of new cases is projected to increase by over 46% by 2040 [3]. The increase is expected primarily among premenopausal patients, in whom, due to the difficulty of early diagnosis, the disease is often detected at an advanced stage [4]. During the last 10 years, BC incidence rates increased by 2% annually among women aged 20 to 29 and 0.2% among women over 30. Young BC patients display different clinical and oncological features compared to older women [5]. The most common BC subtype in young women is hormone receptor-positive (HR+) BC, accounting for two-thirds of all BC cases [6]. HR+ BC is defined as a tumor expressing estrogen receptor (ER) and/or progesterone receptor (PR). Young HR+ BC patients have a higher risk of cancer-related death than their older counterparts [7]. Women with HR+ disease benefit from hormonal agents with or without suppression of ovarian function. The most common endocrine therapies are tamoxifen and aromatase inhibitors (AIs) combined with gonadotropin-releasing hormone agents (GnRH). Endocrine therapies, according to the most recent trials, should be administered for up to 5 to 10 years to reduce recurrence and mortality [8,9]. However, in younger patients, oncological advantages of prolonged treatment must be balanced with compliance with therapy. Younger age is associated with lower adherence [10], with approximately one-third of patients younger than 40 either not initiating or prematurely discontinuing hormonal therapy (HT) [11]. This could be one of the reasons for the overall inferior survival in this group [12,13]. Therefore, it is fundamental to ensure oncofertility counseling and an adequate follow-up in all young BC patients at the time of cancer diagnosis. Recently, the POSITIVE trial confirmed that interrupting HT in stage I–III HR+ BC premenopausal women to allow them to conceive does not increase the short-term risk of recurrence [14]. This pivotal study is changing clinical practice since more patients are now motivated to undergo fertility preservation (FP) procedures to pursue pregnancy. Currently, the most used FP method is oocyte or embryo cryopreservation. The preferred option for most women is oocyte cryopreservation (OC) due to the necessity of a male partner for embryo cryopreservation [15]. Both oocyte and embryo cryopreservation require 2 weeks of controlled ovarian stimulation (COS) with gonadotropins [15].

Normally, it is advised in young women with cancer needing chemotherapy due to associated gonadotoxicity [16]; however, other cancer treatments, including HT, may also have a potential impact on reproductive health.

Given the tendency to postpone motherhood for socioeconomic reasons and the long duration of endocrine therapies in BC patients, it is crucial to consider FP options even for those not receiving chemotherapy, in order to provide them with the opportunity to make informed decisions about their reproductive future. FP is not only a medical but also an emotional decision, often accompanied by distress and shaped by individual needs, values, and life plans [17,18].

This study’s aim is to present data on FP in a cohort of HR+ BC patients candidate to HT alone, with the primary endpoints of oocyte retrieval after COS and subsequent pregnancy rates.

## 2. Materials and Methods

### 2.1. Patients

**Figure 1:**
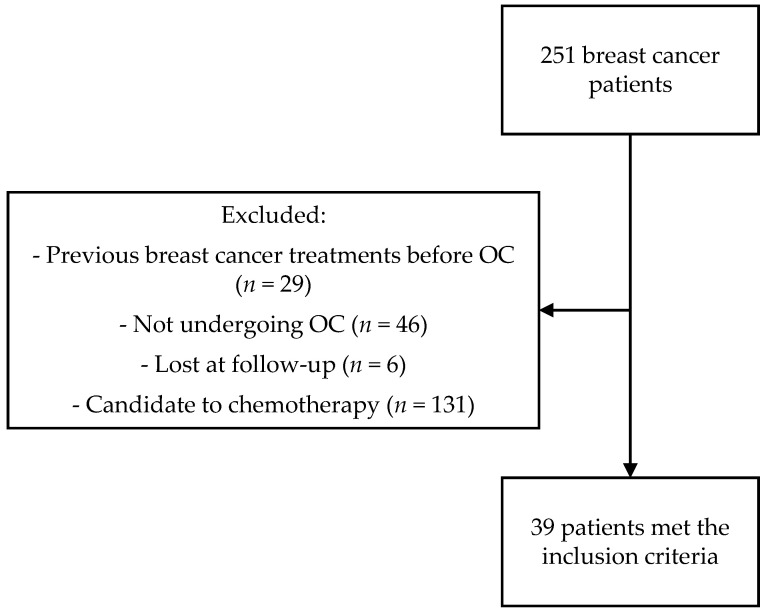
Flowchart of patient selection. OC: oocyte cryopreservation.

This is a single-center prospective cohort study evaluating the data of BC patients receiving FP counseling from 2012 to 2024 at the Oncofertility Unit of San Raffaele Hospital, Milan, Italy. Patients were referred to our Clinic to receive counseling on FP if deemed eligible by their treating physician, i.e., fertile age, adequate performance status and possibility to postpone medical treatment for at least 2 weeks to undergo the procedure. All patients signed an informed consent form, previously approved by the ethics committee, on their first visit to our center. The inclusion criteria for this study were a new diagnosis of BC in patients candidate to endocrine therapy with or without local radiotherapy and OC. Patients were included irrespective of the phase of menstrual cycle, as random-start COS protocols have shown to be as effective as conventional stimulation cycles in cancer patients [19]. Patients receiving chemotherapy or other treatment (i.e., HT) before OC, patients lost at follow-up after initial evaluation, patients candidates to chemotherapy (both neoadjuvant and adjuvant) and patients not pursuing OC were excluded. A flow diagram for patient selection is shown in Figure 1.

All patients were evaluated within 24 h from their first referral in the Oncofertility unit by a team of experts in assisted reproductive techniques (ARTs) and gynecological oncologists. Evaluations occurred at two key points: prior to the initiation of cancer treatment, to provide counseling and FP options, and after the completion of oncological treatment.

Thanks to the results of the POSITIVE trial [14,20,21], some patients in our cohort were allowed to temporarily interrupt HT to have a pregnancy. Patients stopped HT after 18–30 months of treatment and underwent a washout period of 3 months before attempting pregnancy. The duration of interruption was at least 2 years to allow for conception, delivery, and if possible, breastfeeding.

Specific psychological support was offered to all patients interested [17]. Before pregnancy, patients underwent radiological assessments to rule out disease recurrence. The choice between spontaneous conception or in vitro fertilization (IVF) was discussed in a multidisciplinary board based on age, menstrual function and medical history of each patient. The typical interval for attempting spontaneous conception was one year in patients with good ovarian reserve. Once pregnant, we offered access to dedicated pregnancy monitoring in the Obstetric Unit of our hospital to attend to the specific needs of these patients. For this study, we reviewed medical records and collected data on age, body mass index, pretreatment antral follicular count, tumor characteristics (grade, ER expression, PgR expression, HER2, Ki-67 proliferation index), presence of BRCA1-2 mutations, stimulation protocols, egg retrieval outcomes, oncological treatment, pregnancy desire, spontaneous conceptions, IVF cycles, and obstetric course.

### 2.2. Stimulation Protocol

According to ESHRE guidelines [22], for the stimulation, we used recombinant Follicle-Stimulating Hormone (FSH), Human Menopausal Gonadotropin (hMG) and long-acting FSH (corifollitropin alfa) in combination with GnRH antagonists depending on the stimulation phase. Nineteen patients (48.7%) were stimulated regardless of the phase of the menstrual cycle, according to the random start protocol, while the remaining started stimulation with the next menstrual period as it was imminent. When the criterion for triggering final oocyte maturation was reached, induction was performed with 10,000 IU purified recombinant hCG or 0.2 mg GnRH-agonist when at least 3 follicles had reached an average diameter of 18 mm. After retrieval, the oocytes were vitrified, and the patients were redirected to oncologists to start the planned treatment. No embryos were frozen as this is not permitted in Italy for FP purposes.

### 2.3. Statistical Analysis

Statistical analyses were performed using IBM SPSS Statistics software, version 28 for Windows (SPSS, Chicago, IL, USA). Frequencies and relative frequencies were calculated for categorical variables. For continuous variables, the mean and standard deviation were reported if the data were normally distributed, while the median and interquartile range were used for non-normally distributed data.

## 3. Results

In total, 251 patients with a BC diagnosis were referred for counseling on FP to the Oncofertility Unit of San Raffaele Hospital between January 2012 and September 2024. Of these, 39 (23.4%) patients had a BC HR+ treated only with HT and/or radiotherapy and underwent COS. The patient characteristics and treatments are listed in Table 1.

The average time from referral to the beginning of the FP procedures was 7 days (range 0–73 days). One patient waited 73 days before starting COS because of their initial reluctance to start HT. However, following consultations at the oncofertility unit and upon learning about the possibility of OC, she decided to proceed with HT. None of our patients discontinued HT prematurely against medical advice due to concern about treatment-associated infertility. The median duration of COS considered from the day of first drug injection until the oocytes pick up was 12.2 days (range 8–18 days). The median number of follicles measuring more than 16 mm at the time of trigger was 11.36 (range 1–55) while the median number of vitrified oocytes was 10.2 (range 1–24). A double-stimulation protocol was offered to 4 patients (10%) with a mean total vitrified oocyte number of 9.5 (range 5–15) in these patients.

An AI (letrozole 5 mg) was administered daily from the start of gonadotropin treatment until the end of the luteal phase in most cases. Three cycles were conducted using a selective ER modulator (tamoxifen) at a dose of 60 mg daily during the ovarian stimulation period. This was because, at that time, the use of letrozole during ovarian stimulation was off-label in Italy.

The median follow-up time was 65.1 months; six patients were lost at follow-up. To date, among 33 patients with adequate follow-up, 15 patients (45.5%) are still under anticancer treatment after a median follow-up time of 30 months (range 1.5–59.6); 6 patients (18.2%) completed HT but do not seek pregnancy (2 patients are single, 4 patients do not want a pregnancy); 12 patients (36.3%) sought pregnancy, of which 4 sought it after the completion of HT and 8 upon the suspension of HT according to the POSITIVE trial. In total, 9 women (75%) conceived and 8 healthy babies were born (1 patient had 2 pregnancies). The reasons for the remaining three patients, who suspended HT according to the POSITIVE trial, for not having pregnancy, were as follows: one patient (3%) was diagnosed with disease relapse after HT interruption and is now under anticancer treatment; two patients thawed their oocytes without achieving pregnancy and attempted another IVF cycle with failed transfer. Among the nine patients who had a pregnancy, four had finished HT (with a median duration of 60 months). Of these, 1 is currently in her second trimester of pregnancy after thawing her cryopreserved oocytes, while three conceived spontaneously, one is currently in her first trimester of pregnancy, and two delivered (one of them had two spontaneous pregnancies). The remaining five out of nine patients who conceived had stopped HT to seek pregnancy, according to the results of the POSITIVE trial and in agreement with their oncologists. After HT discontinuation, all these patients thawed their oocytes: three had a pregnancy with a live birth, while two patients did not have pregnancy, so one of them attempted a new IVF achieving pregnancy with a live birth, while the other one had a subsequent spontaneous pregnancy.

A total of eight patients thus suspended HT according to POSITIVE study, with one disease relapse and five babies born.

**Figure 2:**
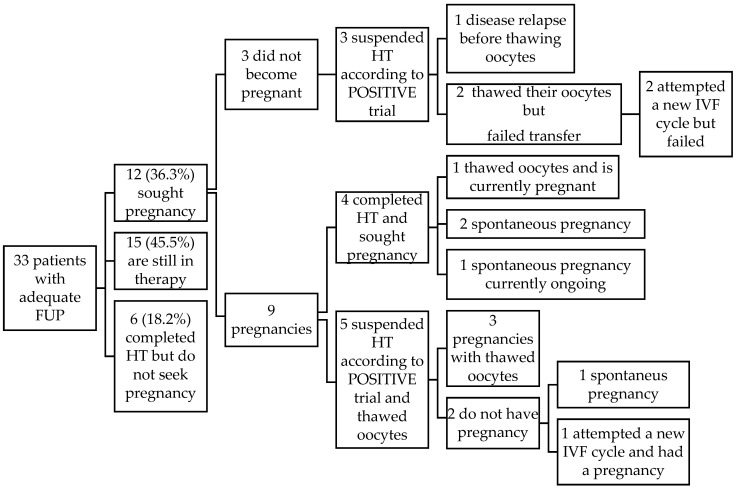
Flowchart of patient follow-up. FUP: follow-up; HT: hormonal therapy; IVF: in vitro fertilization.

Among the nine patients achieving pregnancy so far (four spontaneously; five after IVF), one patient had two spontaneous pregnancies that led to live births, so there were a total of eight live births. The mean age of patients at the time of delivery was 38 years (range 32–41). The gestational age at delivery averaged 38 weeks (range 34–41), with only one case of preterm delivery at 34 weeks. The mean weight of the infants at birth was 2532 g (range 1300–3280). Five pregnancies had a regular obstetric course, one patient had placenta previa, and one patient had Intrauterine Growth Restriction (IUGR); two pregnancies are ongoing without complications. Six patients breastfed, of which three patients needed to supplement breastfeeding with formula milk; one patient failed to breastfeed. Figure 2 depicts the flow-chart of patients’ follow-up. Table 2 reports data on the vitrified oocyte use and IVF attempts of the 12 patients with a desire to become pregnant.

## 4. Discussion

BC is the most common malignancy in young women, and the importance of counseling and access to FP procedures is well established. Our study highlights a specific scenario in which FP may be overlooked—when patients are not scheduled to receive gonadotoxic treatments. This absence may lead to a false sense of reassurance among clinicians. We emphasize the importance of considering FP even in BC patients who are candidates for HT alone. There is paucity of data in the literature on this indication.

Our study is the first to present data on FP outcomes in the specific population of HR+ BC patients undergoing endocrine therapy alone. With a median follow-up of 65.1 months and *n* = 12/33 women attempting conception at a mean age of 38, the proportion of women with successful conception was 75% (*n* = 9/12). Of note, the rate of spontaneous conception was 33.3% (*n* = 4/12), while 41.7% (*n* = 5/12) of women recurring to thawing of cryopreserved oocytes/IVF were successful. This conception rate is higher than what has been reported in the literature, with ranges from 50% to 65% [23,24]. This could be due to the young age of the patients (average 35 years at the time of OC), good ovarian reserve, and also strong patient motivation fostered through counseling and follow-up, which may have positively influenced the return rate.

Considering that, in the general population, the spontaneous pregnancy rate at age 38 is approximately 10–15%, and the success rate of IVF is around 20–30%, these findings suggest that FP performed at an earlier age upon BC diagnosis is highly favorable in this patient population [25]. Additionally, 45.5% of patients in our cohort were still undergoing anticancer treatment after a mean follow-up of 26 months, highlighting the relevance of the POSITIVE trial. These patients may directly benefit from the trial’s outcomes, particularly in relation to fertility planning and management. This may suggest that the chance to preserve fertility may support treatment adherence by aligning medical care with patients’ personal and reproductive goals [18,26]. The possibility of future motherhood plays a key role in many young women’s sense of identity and emotional balance, highlighting the importance of addressing fertility-related concerns as part of comprehensive cancer care [27,28,29]. The primary consideration for FP in BC is oncological safety. Data concerning the safety of COS in patients with HR+ BC are available. Given that BC is a hormonally driven malignancy, concerns have historically been raised regarding the potential risks associated with COS. In a recent meta-analysis and systematic review [30] evaluating the safety of hormonal stimulation in young BC women before starting anticancer treatments, data demonstrate that COS can be performed without negatively impacting patients’ prognosis. Compared to women who did not receive FP before starting treatments, those who underwent COS had a reduced risk of recurrence and mortality. Additionally, no detrimental effect of COS was reported on event-free survival (EFS). A similar outcome in terms of EFS was observed in women with HR+ BC who underwent COS. Women exposed to ART following completion of anticancer treatments also showed better outcomes in terms of recurrence ratio and EFS [30].

In our cohort, only one patient experienced a relapse 36 months after COS. After 26 months of therapy, in agreement with her oncologist, she discontinued HT and tried to conceive spontaneously. Nine months later, she came to our unit to use her cryopreserved oocytes; however, before oocyte thawing, a new imaging evaluation was requested, which revealed a disease relapse. Many oncologists and patients are also concerned about the safety of pregnancy after BC [31]. Since BC is a hormone-driven tumor and considering the surge in female hormones during pregnancy, there is concern that pregnancy could increase patients’ risk of recurrence [32]. However, retrospective data have demonstrated the safety of pregnancy after BC, even in patients with HR+ tumors [33,34,35]. A systematic review and meta-analysis from Lambertini et al. [33] showed no detrimental effect of pregnancy after BC irrespective of tumor characteristics, previous treatment, pregnancy outcome, timing of pregnancy after BC, and BRCA status. Compared with patients with BC without a subsequent pregnancy, those with a post-treatment pregnancy showed better disease-free survival and overall survival [33]. Further data about the safety of attempting pregnancy in HR+ BC come from the results POSITIVE trial [14], a prospective trial designed to address the safety of temporary interruption of HT to attempt pregnancy in HR+ BC patients. In the first analysis, 44 BC events were observed during 1638 patient-years of follow-up. The 3-year cumulative incidences of BC events and distant recurrences were, respectively, 8.9% and 4.5%, similar to those observed in the control cohort. These results suggest that a temporary interruption of HT to attempt pregnancy does not worsen BC prognosis in the short term [14].

At least one pregnancy occurred in 74% of patients enrolled in the trial, with a cumulative incidence of pregnancy at 1 and 2 years from enrollment of 53.6% and 70.5%, Respectively [20]. A clear association between young age and a higher cumulative incidence of pregnancy was observed at both time points. Ovarian stimulation for embryo/oocyte cryopreservation soon after BC diagnosis was performed in 36% of patients, while 43% of patients underwent ART procedures after enrollment in the study. When evaluating the association between ART use and pregnancy, cryopreserved embryo transfer was the only modality associated with a higher chance of pregnancy [20].

Given that FP procedures are safe in BC patients, another issue deserving consideration is the clinical indication. In BC patients, the challenge extends beyond the gonadotoxic effects of cytotoxic chemotherapy. For women with HR+ BC, the recommended 5 to 10 years of adjuvant endocrine therapy often results in significant delays in family planning, further underscoring the importance of timely FP counseling and intervention. In a study of premenopausal women who received chemotherapy followed by tamoxifen treatment, those on tamoxifen had higher rates of amenorrhea 1 to 2 years after chemotherapy [36]. However, by the third year, there was no significant difference in the return of menses, indicating that ovarian suppression due to tamoxifen is temporary and reversible [36]. Additionally, other research has shown that tamoxifen alone, without prior chemotherapy, did not affect the average age of menopause onset, suggesting that tamoxifen is unlikely to substantially speed up ovarian aging [37]. Even if tamoxifen did not directly impact on ovarian reserve, the duration of HT may impact negatively both on ovarian reserve and on adherence to anticancer therapy. AIs (i.e., letrozole), are less frequently used in premenopausal women; hence, their impact on fertility is mostly unknown. It has been shown that a desire for pregnancy can influence patients’ decisions about treatment: Llarena et al. [38] reported that fertility concerns were only second to side effects as the most common stated reason for discontinuation in a cohort of BC patients receiving tamoxifen. During interviews with 88 patients who did not initiate or discontinued tamoxifen, 35% stated that fertility concerns primarily influenced their decision, and 9% felt inadequately counseled on FP [38]. In a study of 643 women with HR+ stage I–III BC one-third indicated that fertility concerns affected HT decisions. Among them, 7% (15/213) did not initiate HT and 33% (70/213) were non-persistent over 5 years of follow-up [39]. Similar data emerged from a survey conducted by the Mayo Clinic Breast Disease Registry [40]. Among the 81 patients who responded to the one-year survey, 53% said they were very concerned about infertility, and among these, 65% reported that this concern greatly influenced their treatment choices. Twenty-two (27%) patients indicated that they had changed their treatment because of its impact on fertility. Among these, 65% shortened the duration of HT to less than 5 years. Additionally, five patients decided not to undergo HT at all [40].

The strength of our study is that, to our knowledge, it is the first one evaluating the opportunity of performing COS before non-gonadotoxic cancer treatments. The main limitations of our study include the small sample size, the single-center design, the absence of a control group and the lack of long-term oncologic outcome analysis. Further prospective multicenter studies on larger cohorts of BC patients or registry-based collaborations would be required to assess the real benefit of performing COS in this group of patients and validate our findings.

## 5. Conclusions

Our study highlights the importance of proceeding with hormonal stimulation and OC in all young patients diagnosed with BC, even those not requiring gonadotoxic chemotherapy. Despite the wide number of studies evaluating the importance of FP in BC patients, there are no studies evaluating the importance of granting access to these procedures even to patients who are not candidates for chemotherapy but only for HT. This represents a critical and emerging area of interest, given the potential impact of FP on adherence to HT, as well as the improved pregnancy outcomes it may yield in this subgroup.

## Figures and Tables

**Table 1:** Patients’ clinical characteristics and oncological treatments.

Characteristics	Value
Patients *n*.	39
Age—Years	
Mean (range)	36 (26–41)
BMI	
Mean (range)	21.19 (16.7–30)
AFC	
Mean (range)	14.23 (5–31)
Genetic predisposition	
BRCA+	2 (5.1%)
BRCA−	30 (76.9%)
Not tested	7 (18%)
Surgery	
Radical	8 (20.5%)
Conservative	31 (79.5%)
Radiotherapy	
Yes	35 (89.7%)
No	4 (10.3%)
HT	
Tamoxifen	8 (20.5%)
AI	3 (7.7%)
LHRH agonist + AI	14 (35.9%)
LHRH agonist + Tamoxifen	14 (35.9%)

BMI: body mass index; AFC: antral follicular count; HT: hormonal therapy; AI: aromatase inhibitor.

**Table 2:** Vitrified oocyte use and IVF attempts in the 12 patients with a desire for pregnancy.

N. pt	Age at COS	N. Tot Oocytes Vitrified	Suspended HT According to Positive	Thawed Oocytes	Pregnancy from Thawed Oocytes	New IVF Cycle	Pregnancy from New IVF Cycle	Spontaneous Pregnancy	Reasons for not Achieving Pregnancy
1	37	7	No	No	-	-	-	Yes	-
2	33	22	No	No	-	-	-	Yes	-
3	37	5	Yes	Yes	No	Yes	No	No	Failed transfer
4	37	5	Yes	No	-	-	-	No	Disease relapse
5	40	5	Yes	Yes	No	Yes	No	No	Failed transfer
6	39	2	No	Yes	Yes (ongoing)	-	-	No	-
7	34	7	Yes	Yes	No	Yes	Yes	No	-
8	26	8	Yes	Yes	Yes	-	-	No	-
9	35	14	Yes	Yes	Yes	-	-	No	-
10	31	4	No	No	-	-	-	Yes (ongoing)	-
11	35	20	Yes	Yes	No	No	-	Yes	-
12	35	20	Yes	Yes	Yes	-	-	No	-

COS: controlled ovarian stimulation; IVF: in vitro fertilization; HT: hormonal therapy.

## Data Availability

The original contributions presented in this study are included in the article. Further inquiries can be directed to the corresponding authors.

## Abbreviations

The following abbreviations are used in this manuscript:

AI	Aromatase Inhibitor
ART	Assisted Reproductive Techniques
BC	Breast Cancer
COS	Controlled Ovarian Stimulation
EFS	Event-Free Survival
ER	Estrogen Receptor
FP	Fertility Preservation
FSH	Follicle-Stimulating Hormone
GnRH	Gonadotropin-Releasing Hormone
hMG	Human Menopausal Gonadotropin
HR+	Hormone Receptor-Positive
HT	Hormonal Therapy
IVF	In Vitro Fertilization
OC	Oocyte Cryopreservation
PR	ProgesteroneRreceptor

## References

[1] Miller K.D., Fidler-Benaoudia M., Keegan T.H., Hipp H.S., Jemal A., Siegel R.L. (2020). Cancer statistics for adolescents and young adults, 2020. CA Cancer J. Clin..

[2] I Numeri Del Cancro 2023. https://www.aiom.it/wp-content/uploads/2023/12/2023_AIOM_NDC-web.pdf.

[3] Heer E., Harper A., Escandor N., Sung H., McCormack V., Fidler-Benaoudia M.M. (2020). Global burden and trends in premenopausal and postmenopausal breast cancer: A population-based study. Lancet Glob. Health.

[4] Cioffi R., Mangili G., Sarais V., Cervini L., Longo V., Bergamini A., Stella Vanni V., Pagliardini L., Candiani M., Papaleo E. (2021). Do Stage and Grade of Malignancy Impact Fertility Preservation in Breast Cancer Patients?. J. Gynecol. Obstet. Hum. Reprod..

[5] Azim H.A., Partridge A.H. (2014). Biology of breast cancer in young women. Breast Cancer Res..

[6] Howlader N., Altekruse S.F., Li C.I., Chen V.W., Clarke C.A., Ries L.A.G., Cronin K.A. (2014). US Incidence of Breast Cancer Subtypes Defined by Joint Hormone Receptor and HER2 Status. JNCI J. Natl. Cancer Inst..

[7] Azim H.A., Michiels S., Bedard P.L., Singhal S.K., Criscitiello C., Ignatiadis M., Haibe-Kains B., Piccart M.J., Sotiriou C., Loi S. (2012). Elucidating Prognosis and Biology of Breast Cancer Arising in Young Women Using Gene Expression Profiling. Clin. Cancer Res..

[8] Early Breast Cancer Trialists’ Collaborative Group (2005). Effects of Chemotherapy and Hormonal Therapy for Early Breast Cancer on Recurrence and 15-Year Survival: An Overview of the Randomised Trials. Lancet.

[9] Bartlett J.M.S., Sgroi D.C., Treuner K., Zhang Y., Ahmed I., Piper T., Salunga R., Brachtel E.F., Pirrie S.J., Schnabel C.A. (2019). Breast Cancer Index and Prediction of Benefit from Extended Endocrine Therapy in Breast Cancer Patients Treated in the Adjuvant Tamoxifen—To Offer More? (ATTom) Trial. Ann. Oncol..

[10] Saha P., Regan M.M., Pagani O., Francis P.A., Walley B.A., Ribi K., Bernhard J., Luo W., Gómez H.L., Burstein H.J. (2017). Treatment Efficacy, Adherence, and Quality of Life Among Women Younger Than 35 Years in the International Breast Cancer Study Group TEXT and SOFT Adjuvant Endocrine Therapy Trials. J. Clin. Oncol..

[11] Rosenberg S.M., Zheng Y., Gelber S., Ruddy K.J., Poorvu P., Sella T., Tamimi R.M., Wassermann J., Schapira L., Borges V.F. (2023). Adjuvant Endocrine Therapy Non-Initiation and Non-Persistence in Young Women with Early-Stage Breast Cancer. Breast Cancer Res. Treat..

[12] Lee H.B., Han W. (2014). Unique Features of Young Age Breast Cancer and Its Management. J. Breast Cancer.

[13] Hershman D.L., Shao T., Kushi L.H., Buono D., Tsai W.Y., Fehrenbacher L., Kwan M., Gomez S.L., Neugut A.I. (2011). Early Discontinuation and Non-Adherence to Adjuvant Hormonal Therapy Are Associated with Increased Mortality in Women with Breast Cancer. Breast Cancer Res. Treat..

[14] Partridge A.H., Niman S.M., Ruggeri M., Peccatori F.A., Azim H.A., Colleoni M., Saura C., Shimizu C., Sætersdal A.B., Kroep J.R. (2023). Interrupting Endocrine Therapy to Attempt Pregnancy after Breast Cancer. N. Engl. J. Med..

[15] Lambertini M., Peccatori F.A., Demeestere I., Amant F., Wyns C., Stukenborg J.-B., Paluch-Shimon S., Halaska M.J., Uzan C., Meissner J. (2020). Fertility Preservation and Post-Treatment Pregnancies in Post-Pubertal Cancer Patients: ESMO Clinical Practice Guidelines. Ann. Oncol..

[16] Cioffi R., Fais M.L., Bergamini A., Vanni V.S., Pagliardini L., Papaleo E., Mangili G., Candiani M. (2022). Ovarian Failure Risk in Post-Pubertal Patients with Cancer: A Prognostic Model. Future Oncol..

[17] Di Mattei V.E., Perego G., Rancoita P.M.V., Taranto P., Carnelli L., Mangili G., Sarais V., Bergamini A., Candiani M. (2020). Psychological Aspects Associated with Fertility Preservation in Oncology: An Exploratory Study. Front. Psychol..

[18] Di Mattei V.E., Taranto P., Perego G., Desimone S., Rancoita P.M.V., Catarinella A., Cioffi R., Mangili G., Vanni V.S., Candiani M. (2023). Identification of Psychological Profiles of Cancer Patients Undergoing Fertility Preservation Counseling. J. Clin. Med..

[19] Cakmak H., Katz A., Cedars M.I., Rosen M.P. (2013). Effective method for emergency fertility preservation: Random-start controlled ovarian stimulation. Fertil. Steril..

[20] Azim H.A., Niman S.M., Partridge A.H., Demeestere I., Ruggeri M., Colleoni M., Saura C., Shimizu C., Saetersdal A.B., Kroep J.R. (2024). Fertility Preservation and Assisted Reproduction in Patients With Breast Cancer Interrupting Adjuvant Endocrine Therapy to Attempt Pregnancy. J. Clin. Oncol..

[21] Partridge A.H., Niman S.M., Ruggeri M., Peccatori F.A., Azim H.A., Colleoni M., Saura C., Shimizu C., Sætersdal A.B., Kroep J.R. (2021). Who Are the Women Who Enrolled in the POSITIVE Trial: A Global Study to Support Young Hormone Receptor Positive Breast Cancer Survivors Desiring Pregnancy. Breast.

[22] Bosch E., Broer S., Griesinger G., Grynberg M., Humaidan P., Kolibianakis E., Kunicki M., La Marca A., Lainas G., Le Clef N. (2020). ESHRE Guideline: Ovarian Stimulation for IVF/ICSI. Hum. Reprod. Open.

[23] Zimmermann A., Perrin J., Deveze C., Saias-Magnan J., Guillemain C., Courbiere B. (2025). Fertility preservation through oocyte or embryo vitrification prior to oncological treatment: A 12-year experience. J. Assist. Reprod. Genet..

[24] Turan V., Oktem O., Bang H., Oktay K.H. (2025). Utilization and Fertility Preservation Outcomes in Women Undergoing Embryo Cryopreservation Before Breast Cancer Treatment: A Meta-Analysis. Clin. Breast Cancer.

[25] Broekmans F.J., Soules M.R., Fauser B.C. (2009). Ovarian Aging: Mechanisms and Clinical Consequences. Endocr. Rev..

[26] Di Mattei V.E., Perego G., Taranto P., Rancoita P.M.V., Maglione M., Notarianni L., Mangili G., Bergamini A., Cioffi R., Papaleo E. (2021). Factors Associated with a High Motivation to Undergo Fertility Preservation in Female Cancer Patients. Front. Psychol..

[27] Di Mattei V.E., Perego G., Taranto P., Mazzetti M., Ferrari F., Derna N., Peccatori F.A., Mangili G., Candiani M. (2023). Psychological Issues in Breast Cancer Survivors Confronted with Motherhood: Literature Review and a Call to Action. Front. Psychol..

[28] Chen M., Wang X., Lan N., Chen Y., Gao Y., Wang J., Wang W., Jiao M., Bai S., Li W. (2025). Prevalence and Impact of Fertility Preservation among Young Women with Breast Cancer. Sci. Rep..

[29] Di Mattei V.E., Perego G., Taranto P., Mazzetti M., Marotta E., Candiani M., Salvatore S. (2021). The Long-Term Effects of Cancer Treatment on Sexuality and Couple Relationships. Fam. Process.

[30] Arecco L., Blondeaux E., Bruzzone M., Ceppi M., Latocca M.M., Marrocco C., Boutros A., Spagnolo F., Razeti M.G., Favero D. (2022). Safety of Fertility Preservation Techniques before and after Anticancer Treatments in Young Women with Breast Cancer: A Systematic Review and Meta-Analysis. Hum. Reprod..

[31] Lambertini M., Di Maio M., Pagani O., Curigliano G., Poggio F., Del Mastro L., Paluch-Shimon S., Loibl S., Partridge A.H., Demeestere I. (2018). The BCY3/BCC 2017 Survey on Physicians’ Knowledge, Attitudes and Practice towards Fertility and Pregnancy-Related Issues in Young Breast Cancer Patients. Breast.

[32] Razeti M.G., Spinaci S., Spagnolo F., Massarotti C., Lambertini M. (2021). How I perform fertility preservation in breast cancer patients. ESMO Open.

[33] Lambertini M., Blondeaux E., Bruzzone M., Perachino M., Anderson R.A., de Azambuja E., Poorvu P.D., Kim H.J., Villarreal-Garza C., Pistilli B. (2021). Pregnancy After Breast Cancer: A Systematic Review and Meta-Analysis. J. Clin. Oncol..

[34] Azim H.A., Kroman N., Paesmans M., Gelber S., Rotmensz N., Ameye L., De Mattos-Arruda L., Pistilli B., Pinto A., Jensen M.-B. (2013). Prognostic Impact of Pregnancy After Breast Cancer According to Estrogen Receptor Status: A Multicenter Retrospective Study. J. Clin. Oncol..

[35] Anderson R.A., Lambertini M., Hall P.S., Wallace W.H., Morrison D.S., Kelsey T.W. (2022). Survival after Breast Cancer in Women with a Subsequent Live Birth: Influence of Age at Diagnosis and Interval to Subsequent Pregnancy. Eur. J. Cancer.

[36] Petrek J.A., Naughton M.J., Case L.D., Paskett E.D., Naftalis E.Z., Singletary S.E., Sukumvanich P. (2006). Incidence, Time Course, and Determinants of Menstrual Bleeding After Breast Cancer Treatment: A Prospective Study. J. Clin. Oncol..

[37] Chien A.J., Duralde E., Hwang R., Tsung K., Kao C.-N., Rugo H.S., Melisko M.E., Esserman L.J., Munster P.N., Cedars M. (2015). Association of Tamoxifen Use and Ovarian Function in Patients with Invasive or Pre-Invasive Breast Cancer. Breast Cancer Res. Treat..

[38] Llarena N.C., Estevez S.L., Tucker S.L., Jeruss J.S. (2015). Impact of Fertility Concerns on Tamoxifen Initiation and Persistence. J. Natl. Cancer Inst..

[39] Sella T., Poorvu P.D., Ruddy K.J., Gelber S.I., Tamimi R.M., Peppercorn J.M., Schapira L., Borges V.F., Come S.E., Partridge A.H. (2021). Impact of Fertility Concerns on Endocrine Therapy Decisions in Young Breast Cancer Survivors. Cancer.

[40] Mannion S., Higgins A., Larson N., Stewart E.A., Khan Z., Shenoy C., Nichols H.B., Su H.I., Partridge A.H., Loprinzi C.L. (2024). Prevalence and Impact of Fertility Concerns in Young Women with Breast Cancer. Sci. Rep..

